# (Ba_0.55_Sr_0.45_)_1−x_La_x_Ti_1.01_O_3_-Bi_0.5_Na_0.5_TiO_3_ Positive Temperature Coefficient Resistivity Ceramics with Low Curie Temperature (~−15 °C)

**DOI:** 10.3390/ma17081812

**Published:** 2024-04-15

**Authors:** Wanlu Xu, Wenwu Wang, Xiaoshan Zhang, Ping Yu

**Affiliations:** College of Materials Science and Engineering, Sichuan University, Chengdu 610064, China; skylar82@163.com (W.X.); www1492@163.com (W.W.);

**Keywords:** PTCR ceramics, low Curie temperature, Bi_0.5_Na_0.5_TiO_3_, BST, sol–gel

## Abstract

Positive temperature coefficient of electrical resistivity (PTCR) materials with low Curie temperature have been paid increasing attention lately. In this study, PTCR materials with a Curie temperature of approximately −15 °C were investigated by La^3+^ doping Ba_0.55_Sr_0.45_TiO_3_ ceramics. It could be expected to meet the requirements of thermal management systems for low-temperature control. In addition, a trace amount of Bi_0.5_Na_0.5_TiO_3_ (BNT) was employed to improve the resistivity and the PTCR performance. A significant PTCR effect was achieved with a high resistivity jump of nearly four orders of magnitude, a high temperature coefficient of ~28.76%/°C, and a narrow transition temperature span of 22 °C in the (Ba_0.55_Sr_0.45_)_0.99875_La_0.00125_Ti_1.01_O_3_-0.0025Bi_0.5_Na_0.5_TiO_3_ ceramics. The PTCR enhancement mechanism of BNT is discussed.

## 1. Introduction

Materials with a positive temperature coefficient of electrical resistance (PTCR) have been widely used as temperature switches or thermistors in overload protectors, self-regulating heaters, starter motors, and resettable fuses [1,2,3]. Their resistances could grow exponentially at *T*_c_ (Curie temperature) or in a narrow temperature span near the phase transformation temperature. Up to now, the switching temperatures of commercial PTCR thermistors are generally higher than 50 °C (which depends on the Curie temperature or the phase transformation temperature of the PTCR materials). However, the demand for low-switching-temperature PTCR materials (<10 °C and even lower) in science technologies and industries is increasing [4]. For example, recently, in-battery heating systems and temperature control systems have been proposed to resist the dysfunctions of lithium-ion batteries that occur in low-temperature environments. As the most promising electrochemical energy storage system, lithium-ion batteries suffer from a series of performance degradations in low-temperature environments (<−10 °C and even lower), such as reduced power performance, significant decreases in charging and discharging efficiency, and reduced cycle life [5,6]. To maintain satisfactory performance for the battery, PTCR thermistors with a low switching temperature (typically −10 °C or −20 °C) are expected to serve as heaters and/or temperature switches [7,8,9,10]. In addition, in the aerospace industry, low-switching-temperature PTCR thermistors (<−10 °C) are also necessary for electronic equipment and thermal control systems applied in low-temperature environments [11]. Accordingly, the research of PTCR materials with low switching temperatures has great potential application [7,10,11,12].

PTCR materials are mainly categorized into three classes: BaTiO_3_ ceramics, V_2_O_3_ ceramics, and polymer composites. Polymer composites, as relatively new materials that are still in development, possess the advantages of low resistivity, large resistance jumps, and an adjustable switch temperature [13]. The PTCR effect of polymer composites stems from the break in the conductive network due to volumetric expansion as the polymer matrix experiences a glassy transition, which leads to a rapid increase in the resistance of polymer composites [14,15,16]. Regrettably, the practical application scenarios of polymer PTCR materials are somewhat restricted. For instance, the issue of PTC reproducibility caused by the irreversible self-aggregation of conductive fillers and the random reconstruction of conductive networks need to be addressed. In addition, most of the low-switching-temperature polymer PTCR materials that have been reported exhibit a strong negative temperature coefficient effect in the non-leap temperature region [17].

The PTCR effect of V_2_O_3_ ceramics can be easily achieved by metal–semiconductor transition in Cr doping (V_1−x_Cr_x_)_2_O_3_ ceramics. Lots of reported research suggests that (V_1−x_Cr_x_)_2_O_3_ ceramics could be a potential option in those environments with temperatures below −30 °C. They show very small electric resistivity before the transition temperature due to their metal conductivity nature. Normally, the resistance jumps of (V_1−x_Cr_x_)_2_O_3_ ceramics could reach to 2~3 orders of magnitude around their transition temperature from −40 °C to −30 °C. However, with the increase in the transition temperature, the resistance jump tends to decrease significantly [18,19]. Furthermore, the PTCR characteristics in (V_1−x_Cr_x_)_2_O_3_ ceramics are always accompanied by large and insurmountable thermal hysteresis phenomena, owing to the residual stresses of the semiconductor phase [18,19].

The PTCR effect in BaTiO_3_ ceramics originates from the ferroelectric–paraelectric phase transition. The resistivity of the semiconducting BaTiO_3_ ceramics would show a remarkable jump during the phase transition. As traditional PTCR materials, BaTiO_3_ ceramics show strong PTCR performance, stable working performance, and an easy adjustment of the *T*_c_ ranging from 50 °C to 135 °C. Generally, the phase transition temperature (*T*_c_) of BaTiO_3_ ceramics could be moved to low temperatures by doping Sr^2+^, Zr^4+^, and/or Sn^4+^ [20]. However, compared with those of *T*_c_ higher than 50 °C, low-*T*_c_ (<10 °C) PTCR BaTiO_3_-based ceramics are seldom reported. A major obstacle is that the PTC performance would be weakened when the Curie temperature is shifted to low temperatures (<25 °C). For instance, the phase transition temperature span (∆T) would widen and the resistance jump would decrease. Encouragingly, some progress has been reported. Yu made significant progress on Ba_1−x_Sr_x_TiO_3_-based PTCR materials with a *T*_c_ of 18 °C by Mn-Y co-doping [21]. The ceramics exhibited a resistivity jump of more than four orders of magnitude, and the resistance temperature coefficient reached 10.7%/°C. Mn improved the surface acceptor state on the grain boundaries, while Y contributed to semiconducting the ceramic in the low-temperature region. Unfortunately, the phase transition temperature span (Δ*T*) was almost 100 °C. For practical use, the value of Δ*T* still needs to be narrowed.

Based on the research that La-doped BaTiO_3_ ceramics show a pronounced PTCR effect [22,23,24], and that a low content of Bi_0.5_Na_0.5_TiO_3_ (BNT) could enhance the PTCR effect by increasing the acceptor density states [25,26,27,28,29,30], in this work, (Ba_0.55_Sr_0.45_)_1−x_La_x_Ti_1.01_O_3_-yBNT PTCR ceramics were designed and fabricated. The aim of this work was to achieve low-Curie-temperature (around −15 °C) PTCR ceramics which possess a large resistance jump, as well as a narrow resistance jump temperature span. The influence of the La^3+^ content on the PTCR performance was investigated experimentally. Furthermore, trace amounts of BNT were introduced to enhance the PTCR performance of the system. Subsequently, the effects of BNT on the PTCR performance of ceramics were investigated systematically.

## 2. Experimental Procedure

### 2.1. Sample Preparation

(Ba_0.55_Sr_0.45_)_1−x_La_x_Ti_1.01_O_3_ ceramic powders (x = 0.001, 0.00125, 0.0025, 0.00375, 0.005) and (Ba_0.55_Sr_0.45_)_0.99875_La_0.00125_Ti_1.01_O_3_-yBi_0.5_Na_0.5_TiO_3_ ceramic powders (y = 0, 0.001, 0.0025, 0.005, and 0.01) were prepared by the sol–gel method. The raw materials (CH_3_COOH)_2_Ba (Aladdin, Shanghai, China, 99%), (CH_3_COOH)_2_Sr (Aladdin, Shanghai, China, 99%), Ti(OC_4_H_9_)_4_ (Chron, Chengdu, China, 98.5%), La(NO_3_)_3_·6H_2_O (Chron, Chengdu, China, 99%), CH_3_COONa·3H_2_O(Chron, Chengdu, China, 99%), and Bi(NO_3_)_3_·5H_2_O (Chron, Chengdu, China, 99%) were weighed according to the designed stoichiometric ratio. Diethanol methyl ether, ethanol acetic acid, and deionized water were used as solvents. All solutions were mixed and stirred until clear and transparent sol formed at 60 °C. The sol solution would turn into gel gradually. The gel was dried in air at 80 °C. The obtained xerogel was carbonized at 350 °C and then calcined at 550 °C, 750 °C, and 950 °C for 3 h, respectively, in air. The obtained fine powder was granulated and pressed into discs of 1.0 cm diameter and 1.0 mm thickness at 10 MPa using polyvinyl alcohol (PVA) as a binder. The discs were calcined to exclude PVA binder at 550 °C. After that, the pellets were sintered at 1330~1405 °C for 3 h in air. Silver paste was sintered on both sides of ceramic pellets at 700 °C for 10 min to form Ag electrodes for electrical measurements.

### 2.2. Measurement and Characterization

The microstructure of the ceramic samples was characterized by using X-ray diffraction (DX-2700, Dandong, China) with Cu K_α_ radiation. The measurements were made at 40 kV and 30 mA with a step size of 0.02 and 1 s/step.

The microscopic morphology of the samples was checked by scanning electron microscopy (Hitachi S-3400N, Tokyo, Japan).

Resistance–temperature (*R*-*T*) characteristics ranging from −40 to 100 °C were conducted by using an Electrometer (Keithley 6517B, Cleveland, OH, USA) with an operating voltage of 5 V in a temperature test chamber with a programmable temperature controller. The resistance–temperature performance of the prepared ceramic samples was obtained in a continuous heating and cooling environment. The resistivity (*ρ*, Ω·cm) can be calculated by the following equation:

(1) $$\rho =\frac{R\pi d^{2}}{4h}$$

where *R* (Ω) is the measured resistance, *d* (cm) is the diameter, and *h* (cm) is the thickness of ceramic pellets.

Capacitor temperature characteristics and impedance characteristics were tested using LCR devices (Tonghui TH2827C, Changzhou, China). The test frequency of the capacitance temperature characteristics was 100 kHz within the temperature range of −40–100 °C. The dielectric permittivity, $\varepsilon_{r},$ can be converted by the following equation:

(2) $$\varepsilon_{r}=\frac{4Ch}{\pi d^{2}\varepsilon_{o}}\;$$

where *C* is the measured capacitance and $\varepsilon_{o}$ is vacuum permittivity. The test voltage of the impedance data was 2 V with the measurement range from 20 Hz to 1 MHz.

## 3. Results and Discussion

**Figure 1:**
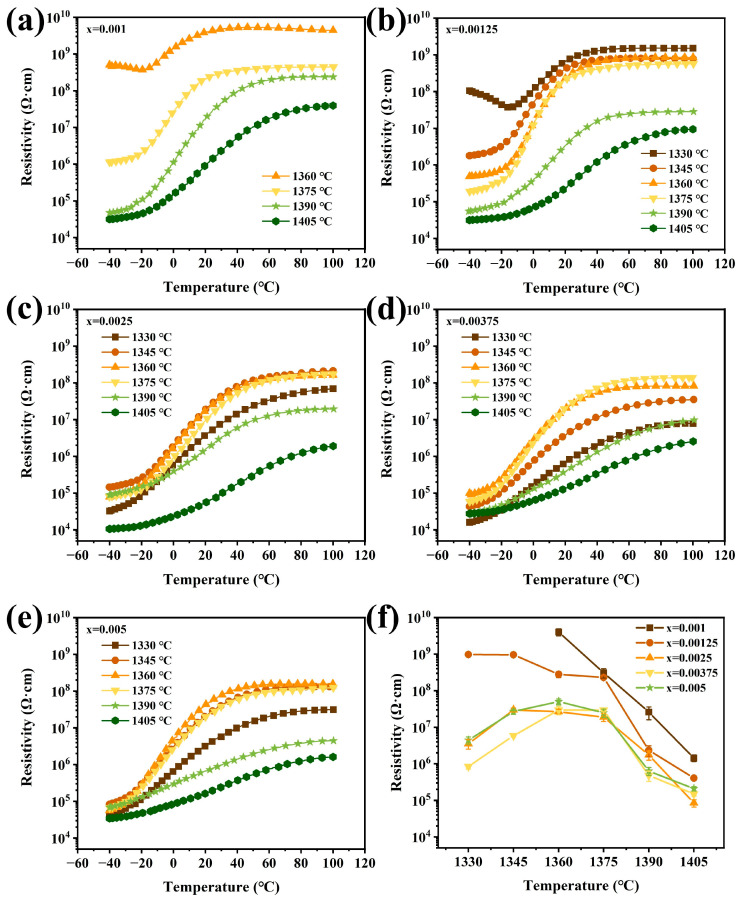
Effects of La content and sintering temperature on the electrical properties of La-doped (Ba_0.55_Sr_0.45_)_(1−x)_La_x_Ti_1.01_O_3_ ceramics (x = 0.001, 0.00125, 0.0025, 0.00375, 0.005): (**a**–**e**) *ρ*-*T* curves for different La concentrations at different temperatures; (**f**) the room temperature resistivity, *ρ*_25_, of the samples sintered at different temperatures.

Figure 1a–e show the temperature-dependent resistivity curves of the prepared (Ba_0.55_Sr_0.45_)_(1−x)_La_x_Ti_1.01_O_3_ ceramics (x = 0.001, 0.00125, 0.0025, 0.00375, 0.005). The ceramic samples were sintered at different temperatures. All samples show a clear PTCR effect. For the samples with x = 0.001 and 0.00125 (see Figure 1a,b), the resistivities decreased significantly with the increasing sintering temperature and the resistivity jumps were enhanced by increasing the sintering temperature. The largest resistivity jumps were achieved in approximately three orders of magnitude when the sintering temperature reached 1390 °C and 1375 °C, respectively. Then, the resistivity jump value dropped with the increasing sintering temperature. For those samples with x = 0.001 sintered at temperatures of 1330 °C and 1345 °C, there were no PTCR effects observed. They showed a white color after sintering, which suggested that higher sintering temperatures were necessary. Comparing the *ρ*-*T* curves shown in Figure 1c–e, the resistivity jump increased with the increasing sintering temperature and then dropped with the further increase in the sintering temperature. The largest resistivity jumps happened in the samples sintered at temperatures of 1375 °C for the samples with x = 0.0025 and 0.00375, and 1360 °C for x = 0.005.

Figure 1f shows the relationship between the sintering temperature and room temperature resistivity of the prepared ceramics. The results indicate that the resistivity of the ceramics decreased notably with the increase in the La content at the same sintering temperature, especially for the samples with x ≤ 0.00375. It is also noticed that for those samples with x = 0.005, the resistivity is larger than that of the samples with 0.0025 and 0.00375 La doped. It is well known that the ionic radius of the La^3+^ ion is 0.116 nm, close to that of the Ba^2+^ ion (0.135 nm) and Sr^2+^ (0.112 nm), and is much larger than that of the Ti^4+^ ion (0.061 nm). As a result, La^3+^ ions are more likely to substitute the A sites in ABO_3_-type structures. Once La substitutes Ba or Sr, defective reactions that may occur, as shown in Equations (3)–(5) [22,23]:

(3) $${La}_{2}O_{3}+2{Ba}_{Ba}^{\times }\to 2{La}_{Ba}^{\cdot }+2e^{\prime }+2BaO+\frac{1}{2}O_{2}$$

(4) $${La}_{2}O_{3}+3{Ba}_{Ba}^{\times }\to 2{La}_{Ba}^{\cdot }+V_{Ba}^{\prime \prime }+3BaO$$

(5) $${2La}_{2}O_{3}+4{Ba}_{Ba}^{\times }+{Ti}_{Ti}^{\times }\to 4{La}_{Ba}^{\cdot }+V_{Ti}^{\prime \prime \prime }+4BaO+TiO_{2}$$

When the content of La ions is small, the A-site substitution of La ions will release electrons, as shown in Equation (3); the conductivity is attributed to a change in doping mechanism, dominated by electronic compensation. Therefore, the resistivity of ceramics will be reduced. However, with the increase in the La^3+^ content, cation vacancies may be produced for the defect reactions, as shown in Equations (4) and (5). Ulteriorly, with the further increasing La content, the cation vacancy compensation gradually dominates the situation, which leads to an increase in resistivity.

Obviously, for the samples with different La contents, there are optimum sintering temperatures for better PTCR performance. These are 1390 °C for the samples with x = 0.001; 1375 °C for x = 0.00125, 0.0025, and 0.00375; and 1360 °C for x = 0.005. The optimum sintering temperature tends to be lower with the increase in the La content. This reveals that La^3+^ can promote sintering and enhance the PTCR performance significantly. These results indicate that the PTCR performance of the samples is closely linked with the sintering temperature and La^3+^ content. In addition, as Ba_1−x_Sr_x_TiO_3_ normally possesses higher resistivity than BaTiO_3_ and its resistivity increases with the increase in the Sr content [31], the La-doped Ba_0.55_Sr_0.45_TiO_3_ ceramics show higher resistivity than the La-doped BaTiO_3_. In our case, the resistivities of all the samples below *T*_c_ are 10^4^–10^5^ Ω·cm. Similar results are reported in reference [21].

**Figure 2:**
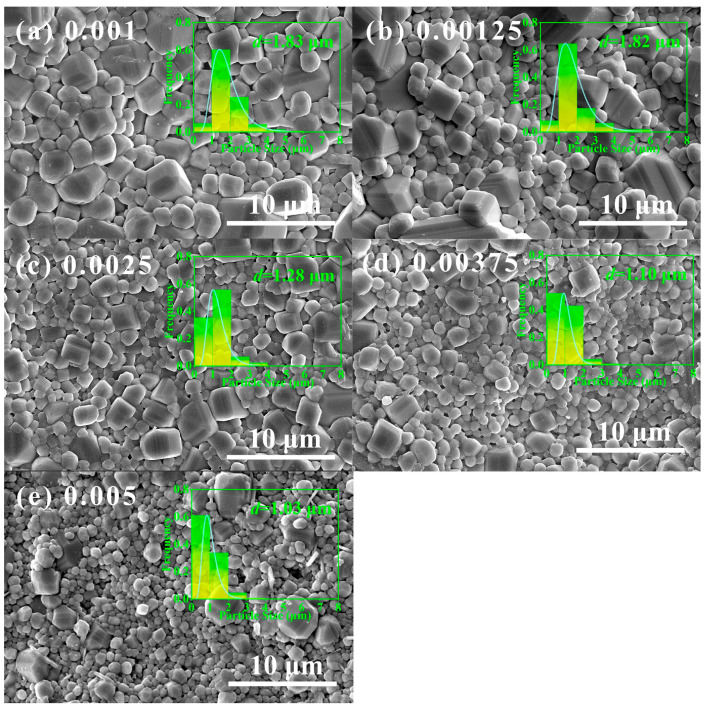
The SEM images of (Ba_0.55_Sr_0.45_)_(1−x)_La_x_Ti_1.01_O_3_ ceramics. (**a**) x = 0.001; (**b**) 0.00125; (**c**) 0.0025; (**d**) 0.00375; (**e**) 0.005.

Figure 2 shows the SEM micrographs of the La-doped Ba_0.55_Sr_0.45_TiO_3_ ceramic samples sintered at their optimum sintering temperatures. It reveals that the La content affected the grain growth of La-doped Ba_0.55_Sr_0.45_TiO_3_ ceramics significantly. The grain size tends to be small with the increase in the La content. Generally, the small grain size would lead to a large resistivity of the ceramics due to a large amount of grain boundaries. As a result, the La content could increase the resistivity of Ba_0.55_Sr_0.45_TiO_3_ ceramics by cation vacancy compensation and inhibiting the growth of grains.

**Figure 3:**
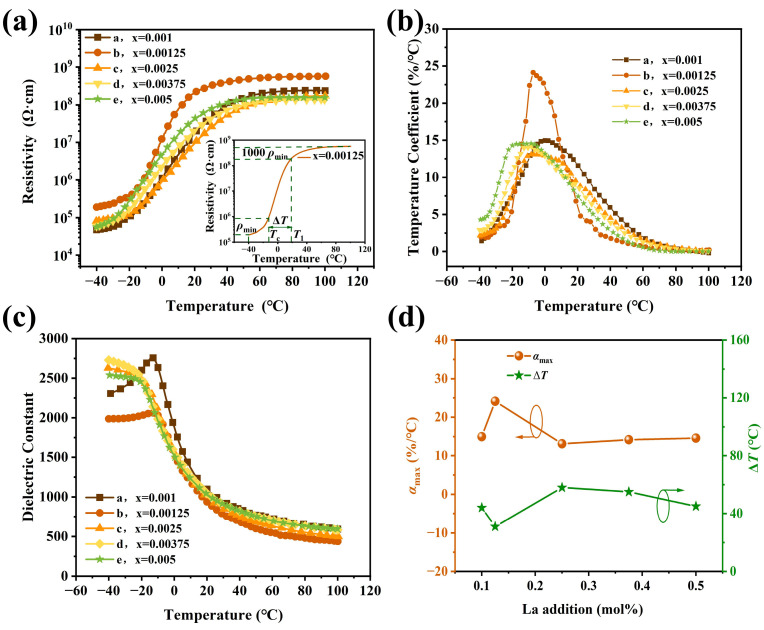
(**a**) *ρ*-*T* curves of ceramics with different x values sintered at optimum sintering temperature. The inset is the *ρ*-*T* curve of x = 0.00125. (**b**) The temperature dependence of resistivity temperature coefficient, *α,* of the samples. (**c**) The *ε*_r_-*T* curves of the ceramic samples with different La contents. (**d**) La content dependence of *α*_max_ and Δ*T* of the samples.

Figure 3a gives the resistivity temperature response of ceramic samples with different La contents. All the ceramics were sintered at their optimum sintering temperatures. In order to evaluate the PTCR performance clearly, here, the *ρ* at −40 °C is defined as *ρ*_min_ (considering the facts that the resistivities increase monotonically with the increase in the temperature before the phase transition in our case); the *T*_c_ of the ceramics is determined by a dielectric constant versus temperature curve of the ceramics in Figure 3c; the phase transition temperature span, Δ*T*, is defined as the temperature span in which resistivity rises from *ρ_T_*_c_ to 1000*ρ_min_*; and the PTC resistivity jump is defined as lg *ρ*_max_/*ρ_min_*.

The results in Figure 3a reveal that the *T*_c_ of the samples is located in the temperature range from −20.36 °C to −12.68 °C, and the resistivity jumps are around three for all samples. The room temperature resistivities of the prepared ceramics are around ~×10^8^ Ω·cm. Furthermore, hardly any NTC phenomena appeared in the tested temperature range and a near-plateau is observed from 20 °C to 100 °C for all samples. Among them, the sample with x = 0.00125 shows the narrowest phase transition temperature span of Δ*T* ~31 °C. As shown in the Figure 3a inset, in the first stage, the resistivity rises mildly from −40 °C to −30 °C. An appreciable increase in electrical resistivity occurs from −30 °C to −13 °C. After that, a more dramatic change starts from −13 °C to 18 °C, when the electrical resistivity increases from 7.36 × 10^5^ Ω·cm to 1.88 × 10^8^ Ω·cm and then reaches the value of 2.53 × 10^8^ Ω·cm at 25 °C. Then, the electrical resistivity increases from 2.53 × 10^8^ Ω·cm to 5.7 × 10^8^ Ω·cm slowly in the temperature range from 25 °C to 100 °C, and the curve almost displays a plateau, which should be helpful in practical applications, where it is expected that PTCR materials keep a constant resistivity or mild NTC effect in resistivity above switch temperature.

The resistivity temperature coefficient, *α_T_*, was calculated from the *ρ*-*T* curve given in Figure 3a as follows:

(6) $$\alpha_{T}=\frac{1}{R_{T}}\frac{dR_{T}}{dT}\times 100\%$$

Here, *R_T_* is the resistance corresponding to the certain temperature of *T*.

The *α_T_*-*T* curves of all samples are shown in Figure 3b. It reveals that all temperature coefficient maximum values of the samples are located between −20 °C and 10 °C. Among them, the sample with 0.00125 La content shows an outstanding high value of 24.12%/°C at −7.24 °C. The La content dependence of *α_T_* and the phase transition temperature span, Δ*T*, are shown in Figure 3d. It suggests that a small proper amount of La doping will improve the resistance temperature response in the PTCR jump interval; however, an excessive amount of La doping will lead to a decrease in the temperature coefficient. The temperature dependence of the dielectric constant of the samples is shown in Figure 3c. The results suggest that the ferroelectric–paraelectric phase transition of the ceramics tended toward relaxation when the La content was higher than 0.00125. As a result, an obvious large phase transition temperature span (Δ*T*) happened in the case of x = 0.0025, 0.00375, and 0.005 (see Figure 3d).

**Figure 4:**
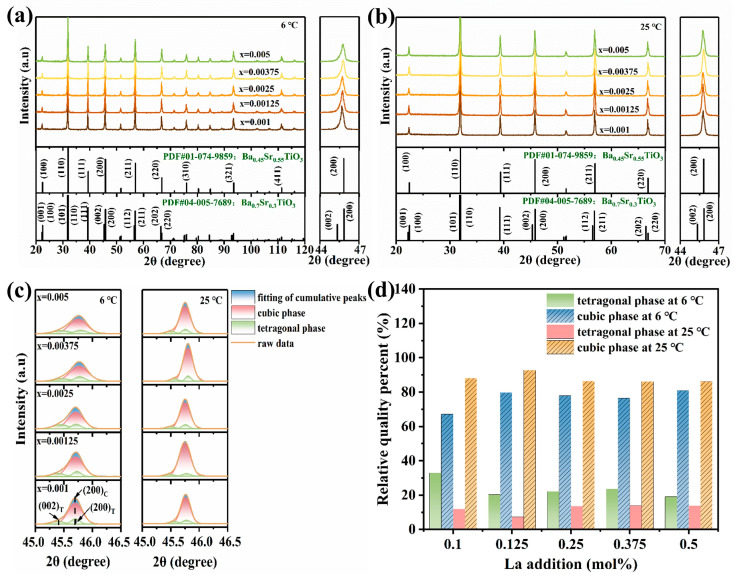
The XRD patterns of the ceramic samples with different La contents at the different temperatures: (**a**) 6 °C and (**b**) 25 °C. (**c**) A close observation in 2θ ranging from 45° to 46.5°. (**d**) Calculated relative quality percentages of tetragonal and cubic phases at 6 °C and 25 °C.

Figure 4 shows the XRD patterns of all samples tested at temperatures of 6 °C and 25 °C. Compared with PDF cards #01-074-9859 and #04-005-7689, all samples showed a single perovskite phase, and no second phase was detected in the tested range. In a close observation, it could be noticed that the diffraction peaks of all the samples showed mild left- shifting with the increase in the La content, as shown in Figure 4a,b. This indicates the slight enlargement of the lattice due to the La entering the Ba_0.55_Sr_0.45_TiO_3_ lattice. Furthermore, the asymmetry of the diffraction peaks (~45°) suggest that the cubic and tetragonal phases coexist in all samples, especially for the samples at 6 °C. They could be fitted by the characteristic peaks of a tetragonal structure and a cubic structure (see Figure 4c). The results reveal that the cubic and tetragonal phases coexisted in all samples at 6 °C, and the tetragonal phase reduced significantly when the temperature reached 25 °C. The phase composition of the samples at the tested temperatures were analyzed by RIR methods. The results are listed in Table 1. It suggests that the ferroelectric–paraelectric phase transition would be near completion at 25 °C. The results are consistent with that of the resistivity–temperature curves in Figure 3a and the dielectric constant–temperature curves in Figure 3c.

To evaluate the performance of the samples clearly, the PTCR performance parameters of the samples in Figure 3a are listed in Table 1. Among them, sample *b* possesses the large resistance jump of ~3.48, the narrowest transition temperature span of ~31 °C, and the largest resistivity at 25 °C.

**Figure 5:**
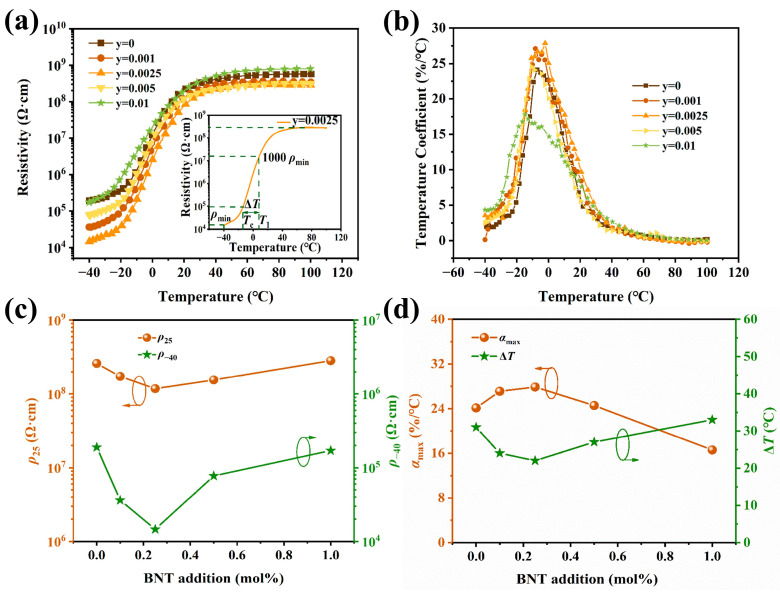
Effects of BNT content on the properties of (Ba_0.55_Sr_0.45_)_0.99875_La_0.00125_Ti_1.01_O_3_-yBNT (y = 0, 0.001, 0.0025, 0.005, 0.01) ceramics sintered at 1375 °C. (**a**) *ρ*-T curves of different y values; the inset is the *ρ*-T curve of y = 0.0025. (**b**) The temperature dependence of the resistivity temperature coefficient α of the samples. (**c**) BNT content dependence of *ρ*_25_ and *ρ*_−40_ of the samples. (**d**) BNT content dependence of α_max_ and Δ*T* of the samples.

Under the consideration that a resistivity as low as possible before the phase transition is expected in practical use, as it would reduce the power consumption of the PTC thermistors, trace Bi_0.5_Na_0.5_TiO_3_ (BNT) was employed to modulate the resistivity of the (Ba_0.55_Sr_0.45_)_(1−x)_La_x_Ti_1.01_O_3_ ceramics (x = 0.00125) in this work.

(Ba_0.55_Sr_0.45_)_0.99875_La_0.00125_Ti_1.01_O_3_-yBNT ceramics (y = 0, 0.001, 0.0025, 0.005, and 0.01) were prepared. Figure 5 shows the resistivity temperature response of the samples sintered at their optimum sintering temperatures. All samples show a similar resistivity temperature response to that in Figure 3a (see Figure 5a).

Overall, the resistivities are reduced by introducing BNT; in particular, the changes in the resistivity before the phase transition are notably larger than that after phase transition, except for samples with y = 0.005 (see Figure 5c). As a result, the PTC jumps of all samples are enhanced. Compared with the curves in Figure 3b, the *α_T_* values of all samples (see Figure 5b) are improved by introducing BNT. The samples with 0.0025 BNT content show the highest value of 27.86%/°C at −2.14 °C.

Figure 5d shows the BNT content dependence of *α_T_* and Δ*T*. The results indicate that proper doping of BNT could increase the temperature coefficient of resistance. The change in *T*_c_ is mild due to the trace BNT amount, even though BNT possess a much higher *T*_c_. As a result, a proper amount of BNT would strengthen the PTC performance of (Ba_0.55_Sr_0.45_)_0.99875_La_0.00125_Ti_1.01_O_3_ ceramics. Similar results are reported in research on high-*T*_c_ (>120 °C) BaTiO_3_-based materials [26,27,28,29,30,32,33,34]. The detailed PTCR performance parameters of the samples are listed in Table 2.

**Figure 6:**
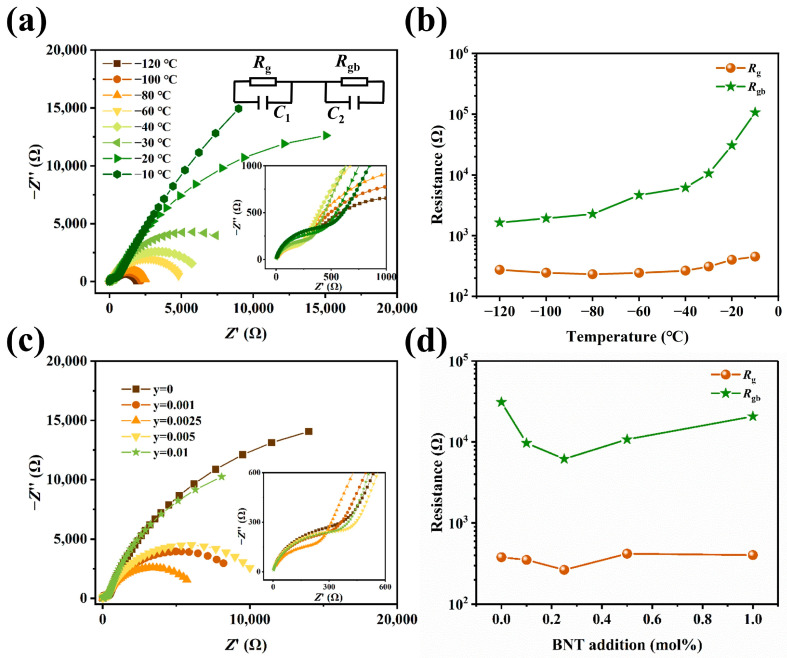
The complex impedance spectra of the (Ba_0.55_Sr_0.45_)_0.99875_La_0.00125_Ti_1.01_O_3_-yBNT ceramics. (**a**) Impedance spectra of (Ba_0.55_Sr_0.45_)_0.99875_La_0.00125_Ti_1.01_O_3_-0.0025BNT ceramic. (**b**) Temperature dependence of *R*_g_ and *R*_gb_ values. (**c**) Impedance spectra of (Ba_0.55_Sr_0.45_)_0.99875_La_0.00125_Ti_1.01_O_3_-yBNT (y = 0, 0.001, 0.0025, 0.005, 0.01) ceramics at −40 °C. (**d**) *R*_g_ and *R*_gb_ of ceramics with different BNT contents at −40 °C.

Impedance spectra are very useful in separating the contribution of different electrical components from whole bulk ceramics, such as grains, grain boundaries, and other regions [32]. Since impedance spectra can usually be analyzed from capacitance data using equivalent circuits for grain boundaries and grain effects, each of these components can be represented by a parallel RC [35,36,37]. In order to understand the influence of BNT on the resistivity of (Ba_0.55_Sr_0.45_)_(1−x)_La_x_Ti_1.01_O_3_ ceramics, the complex impedance spectra of the samples were measured at different temperatures. Figure 6 gives the impedance spectra of the samples. An equivalent circuit diagram for each ceramic grain could be fitted by the impedance spectra, where *R*_g_ and *R*_gb_ denote grain resistance and grain boundary resistance, respectively [38]. Figure 6a shows the impedance spectra of sample No. 3 in Table 2 measured at different temperatures. As shown in Figure 6a, the impedance spectra are all able to fit two semicircular arcs, and the *R*_g_ and *R*_gb_ were estimated from the equivalent circuit diagram. The calculated values of grain and grain boundary resistance versus temperature from Figure 6a is shown in Figure 6b. For impedance (Z’’) and modulus (M’’) spectroscopic plots at different temperatures for ceramic samples, see Figure S1.

Compared with the ceramics without BNT, the *R*_gb_ was reduced by nearly two orders of magnitude. Also, the *R*_gb_ was more than one order of magnitude greater than the *R*_g_. The *R*_g_ changed mildly in the investigated temperature region. Different from *R*_g_, *R*_gb_ has a significant growth rate from −20 °C, which is consistent with the resistivity change in Figure 5a. It suggests that the anomalous increase in resistance is mainly related to the change in *R*_gb_ values. Clearly, *R*_gb_ plays an important role in the PTCR phenomenon. Figure 6c shows the impedance profiles of different contents of BNT at −40 °C. The results reveal that the trace BNT could affect both *R*_g_ and *R*_gb_ (see Figure 6d), and, like the results of Figure 6b, the resistivity of the ceramics was determined by the *R*_gb_ due to it being more than one order of magnitude greater than the *R*_g_.

According to the Heywang model, polycrystalline ceramic materials would have a two-dimensional surface host state on the grain surface which traps electrons from the vicinity and induces the creation of a depletion layer, which is the origin of the grain boundary resistance. The height of the potential barrier in the depletion layer can determine the magnitude of the PTCR effect.

The height of the potential barrier (Φ) of ceramics can be calculated according to the following equation [38]:

(7) $$\rho_{gb}=\beta \rho_{g}exp\left(\frac{e\Phi }{kT}\right)$$

where β is a factor of geometrical configuration, *e* is the electric charge, and *k* is the Boltzmann constant. Equation (7) can be further calculated to give the following equation:

(8) $$ln\left(\rho_{gb}\right)=ln\left(\beta \rho_{g}\right)+\frac{e\Phi }{kT}$$

**Figure 7:**
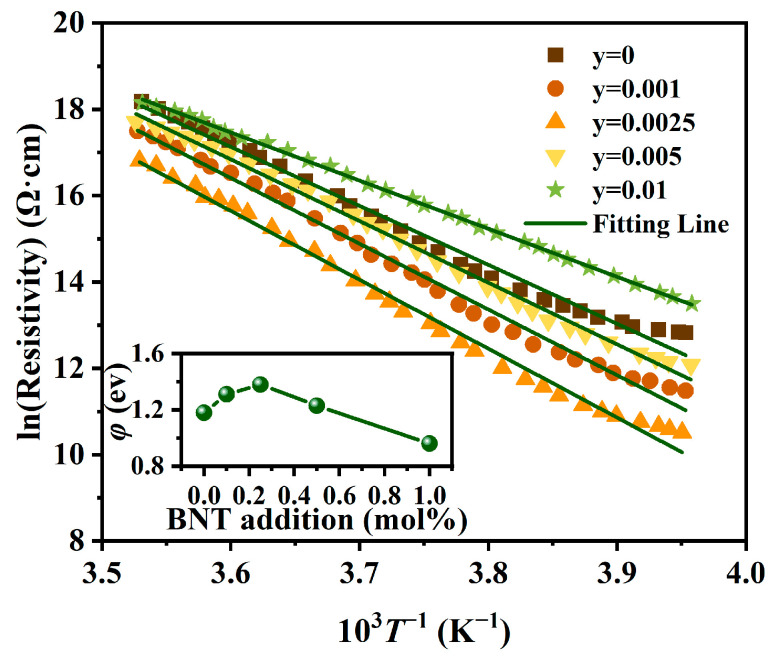
The curve of *lnρ_gb_* against 10^3^*T*^−1^ from −20 °C to 10 °C (estimated potential barrier height shown in inset) for the (Ba_0.55_Sr_0.45_)_0.99875_La_0.00125_Ti_1.01_O_3_-yBNT (y = 0, 0.001, 0.0025, 0.005, 0.01) ceramics sintered at 1375 °C.

In this equation, $ln\left(\rho_{gb}\right)$ and *T*^−1^ lead to an approximate linear relationship near the Curie temperature; thus, we plot *lnρ*_gb_ versus 1000/*T* to calculate the height of the potential barrier from −20 °C to 10 °C for different BNT concentrations, as shown in Figure 7.

The calculated results indicate that the potential barrier height increased to the highest value of 1.4 eV at 0.0025 BNT, and then dropped with the increasing BNT content. This implies that the proper trace BNT content could increase the potential barrier height by modulating the density of acceptors and the donor concentration of ceramic grains. The addition of BNT to (Ba_0.55_Sr_0.45_)_0.99875_La_0.00125_Ti_1.01_O_3_ ceramics tends to produce two main new defects: ${Bi}_{Ba}^{•}$ and ${Na}_{Ba}^{\prime }$ (in the case of Ba, Sr can replace Ba equally). During the sintering process of (Ba_0.55_Sr_0.45_)_0.99875_La_0.00125_Ti_1.01_O_3_-yBNT ceramics, the acceptor defects tend to segregate to grain boundaries, and donor defects are often not considered to segregate to grain boundaries [34]. Also, Na displays the preferred evaporation compared to Bi [33]. Therefore, the BNT in (Ba_0.55_Sr_0.45_)_0.99875_La_0.00125_Ti_1.01_O_3_ ceramics mainly affects the potential barrier height through the volatilization of the element and the adjustment of the Ba/Ti ratio at the grain boundary.

Additionally, the easy volatilization characteristics of Bi^3+^ and Na^+^ as well as ionic vacancy migration could promote the more even Sr distribution in (Ba_0.55_Sr_0.45_)_0.99875_La_0.00125_Ti_1.01_O_3_-yBNT ceramics [39]. As a result, the trace addition of BNT mitigated the phenomenon of diffused phase transition resulting from heavily doped Sr^2+^ in (Ba_0.55_Sr_0.45_)_(1−x)_La_x_Ti_1.01_O_3_ ceramics. This leads to the acceleration of the phase transition and the increase in the resistance temperature coefficient.

**Figure 8:**
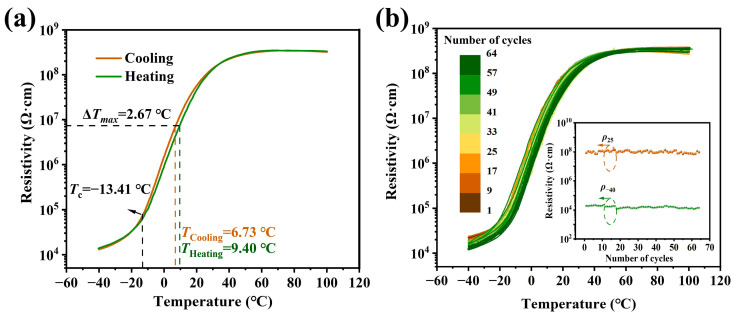
(**a**) The heating and cooling curves of the (Ba_0.55_Sr_0.45_)_0.99875_La_0.00125_Ti_1.01_O_3_-0.0025BNT ceramic. (**b**) The reproducibility of *ρ*-T curves for the (Ba_0.55_Sr_0.45_)_0.99875_La_0.00125_Ti_1.01_O_3_-0.0025BNT ceramic; the inset is *ρ*_−40_ and *ρ*_25_ changes with cycling test curves.

Figure 8 shows the cooling and heating resistivity temperature responses and the reproducibility of the PTCR performance of the (Ba_0.55_Sr_0.45_)_0.99875_La_0.00125_Ti_1.01_O_3_-0.0025BNT ceramic. The PTCR jumps are almost 4.30 and the thermal hysteresis, ∆*T*_max_, is around −2.67 °C, resulting from the inevitable thermal hysteresis in the phase transition of the ceramic (see Figure 8a). For a practical case, a small thermal hysteresis value is expected. Figure 8b shows that after 64 continuous rounds of testing for more than 30 days, the *T*_c_ and the PTCR jump keep values of −13.41 °C and ~4.30 stably. The room temperature resistivity’s changing ratio is less than 15% in the first 10 days and then tends to stabilize as time passes. This suggests that the PTCR characteristics of the sample show good stability, which is the prerequisite of temperature-controlling switch applications.

## 4. Conclusions

In summary, (Ba_0.55_Sr_0.45_)_(1−x)_La_x_Ti_1.01_O_3_-yBNT PTCR ceramics with low *T*_c_ (~−15 °C) were investigated. Proper La element doping could drop the *T*_c_ of Ba_0.55_Sr_0.45_TiO_3_ ceramics to below 0 °C and shows satisfactory PTCR performance. In our doping region, the (Ba_0.55_Sr_0.45_)_(1−x)_La_x_Ti_1.01_O_3_ ceramics showed a resistivity jump of around three at Curie temperatures. The (Ba_0.55_Sr_0.45_)_0.99875_La_0.00125_Ti_1.01_O_3_ ceramics possess a resistivity jump of 3.48 in a narrow temperature span of ~31 °C, and the *T*_c_ is −12.68 °C. Meanwhile, specimens show relatively low resistivity below *T*_c_ as well as large PTC effects by co-doping small amounts of BNT. In the present work, it was shown that trace amounts of BNT can reduce the resistivity and increase the temperature coefficient of (Ba_0.55_Sr_0.45_)_0.99875_La_0.00125_Ti_1.01_O_3_ ceramics significantly.

The ceramics of (Ba_0.55_Sr_0.45_)_0.99875_La_0.00125_Ti_1.01_O_3_-0.0025BNT show remarkably large temperature coefficients (α*_T_* = 27.86%/°C) at −2.14 °C and a high resistivity jump lg(*ρ*_max_/*ρ*_min_) up to 4.30 in a narrow temperature span of 22 °C. Compared with the reported low-switch-temperature PTCR materials [18,19,20,21], (Ba_0.55_Sr_0.45_)_0.99875_La_0.00125_Ti_1.01_O_3_-0.0025BNT PTCR ceramics exhibit outstanding comprehensive PTCR performance. In particular, the proper *T*_c_ (~−15 °C), the acceptable narrow temperature span (Δ*T*, 22 °C), the sufficiently large resistivity jump value (~4.3), the small thermal hysteresis value (−2.67 °C), and the satisfactory stability make them potential thermistor materials to be applied to internal heating control systems of lithium-ion batteries.

## Figures and Tables

**Table 1:** Compositions and performance parameters of the (Ba_0.55_Sr_0.45_)_(1−x)_La_x_Ti_1.01_O_3_ ceramic samples.

Sample	x	*T*_sintering_ (°C)	*ρ*_25_ (Ω·cm)	*T*_1_ (1000 *ρ*_min_) (°C)	*T*_c_ (°C)	Δ*T* (°C)	lg*ρ*_max_/*ρ*_min_
a	0.001	1390	2.60 × 10^7^	30.36	−13.71	44	3.71
b	0.00125	1375	2.53 × 10^8^	18.31	−12.68	31	3.48
c	0.0025	1375	2.66 × 10^7^	42.49	−15.27	58	3.32
d	0.00375	1375	3.10 × 10^7^	35.59	−19.57	55	3.37
e	0.005	1360	5.02 × 10^7^	24.88	−20.36	45	3.45

**Table 2:** Performance parameters of the (Ba_0.55_Sr_0.45_)_0.99875_La_0.00125_Ti_1.01_O_3_-yBNT ceramics.

Sample	y	*ρ*_25_ (Ω·cm)	*T*_1_ (1000*ρ*_min_) (°C)	*T*_c_ (°C)	Δ*T* (°C)	lg*ρ*_max_/*ρ*_min_
1	0	2.53 × 10^8^	18.31	−12.68	31	3.48
2	0.001	1.73 × 10^8^	9.45	−14.19	24	4.01
3	0.0025	1.18 × 10^8^	8.17	−13.41	22	4.30
4	0.005	1.15 × 10^8^	13.92	−12.96	27	3.61
5	0.01	2.83 × 10^8^	18.26	−15.10	33	3.67

## Data Availability

The raw data supporting the conclusions of this article will be made available by the authors on request.

## Supplementary Materials

The following supporting information can be downloaded at https://www.mdpi.com/article/10.3390/ma17081812/s1.
