# Supplementary material for: (Ba0.55Sr0.45)1−xLaxTi1.01O3-Bi0.5Na0.5TiO3 Positive Temperature Coefficient Resistivity Ceramics with Low Curie Temperature (~−15 °C)
Source: Materials (Basel). 2024 Apr 15;17(8):1812. doi: 10.3390/ma17081812 (PMC11051436; doi:10.3390/ma17081812)
Supplement: Supplementary file 1 [file materials-17-01812-s001.zip › materials-2944196-supplementary.pdf]

Supplementary Materials

$(Ba_{0.55}Sr_{0.45})_{1-x}La_xTi_{1.01}O_3$-$Bi_{0.5}Na_{0.5}TiO_3$ Positive Temperature Coefficient Resistivity Ceramics with Low Curie Temperature (~−15 °C)

Wanlu Xu, Wenwu Wang, Xiaoshan Zhang and Ping Yu *

College of Materials Science and Engineering, Sichuan University, Chengdu 610064, China; skylar82@163.com (W.X.); www1492@163.com (W.W.); zhills0206@hotmail.com (X.Z.)

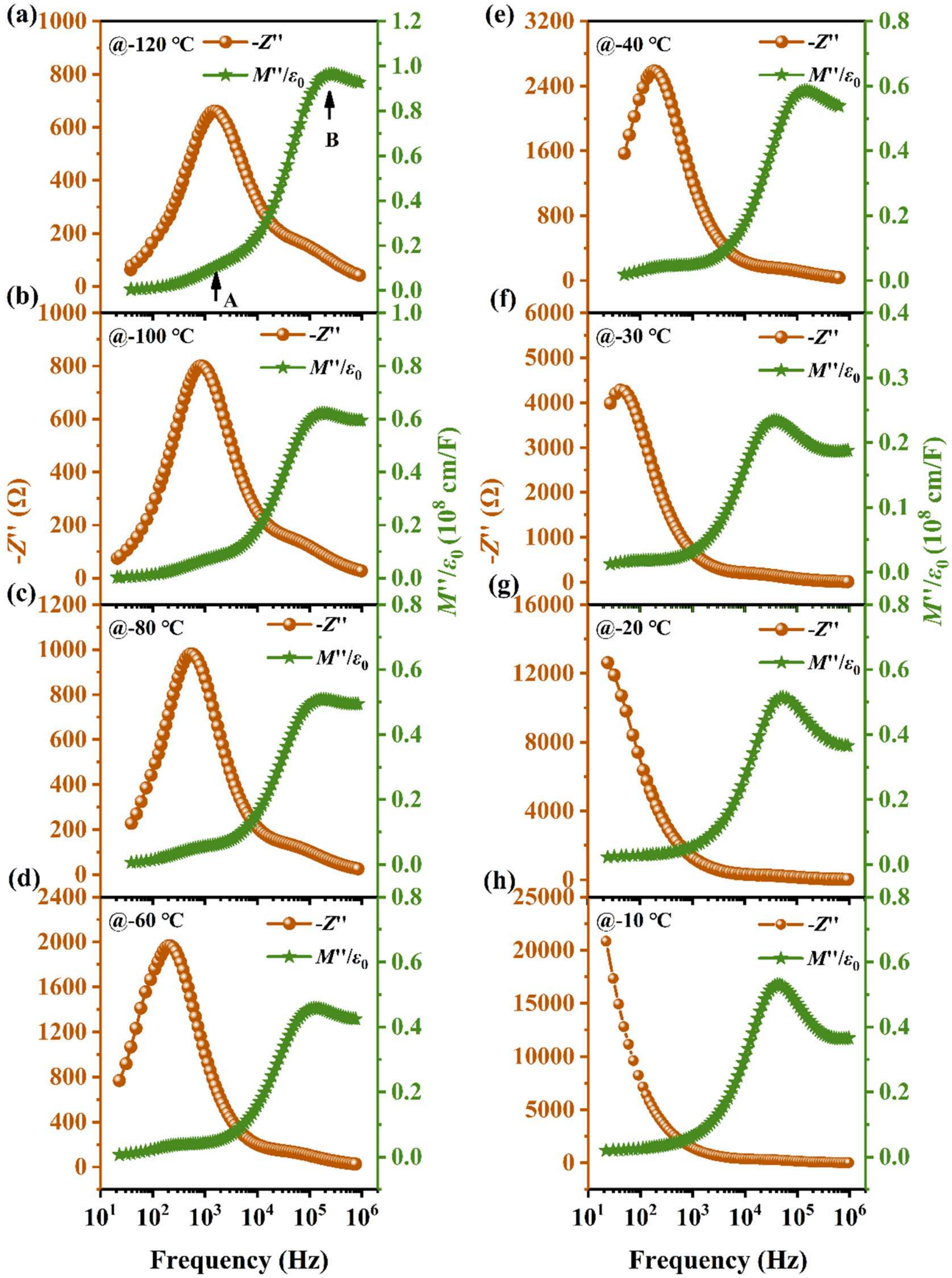

Figure S1. Impedance Z''and modulus M'' spectroscopic plots at different temperatures for $(Ba_{0.55}Sr_{0.45})_{0.99875}La_{0.00125}Ti_{1.01}O_3$ -0.0025BNT ceramic at different temperatures.
